# PP6 negatively modulates LUBAC-mediated M1-ubiquitination of RIPK1 and c-FLIP_L_ to promote TNFα-mediated cell death

**DOI:** 10.1038/s41419-022-05206-9

**Published:** 2022-09-07

**Authors:** Guowei Wu, Dekang Li, Wei Liang, Weimin Sun, Xingxing Xie, Yilun Tong, Bing Shan, Mengmeng Zhang, Xiaojuan Lu, Junying Yuan, Ying Li

**Affiliations:** 1 Interdisciplinary Research Center on Biology and Chemistry, Shanghai Institute of Organic Chemistry, Chinese Academy of Sciences, 100 Haike Road, PuDong District, 201210 Shanghai, China; 2 University of Chinese Academy of Sciences, 100049 Beijing, China

**Keywords:** Necroptosis, Apoptosis

## Abstract

Activation of TNFR1 by TNFα induces the formation of a membrane-associated, intracellular complex termed complex I. Complex I orchestrates a complex pattern of modifications on key regulators of TNF signaling that collectively determines the cell fate by activating pro-survival or executing cell death programs. However, the regulatory mechanism of complex I in cell-fate decision is not fully understood. Here we identify protein phosphatase-6 (PP6) as a previously unidentified component of complex I. Loss of PP6 protects cells from TNFα-mediated cell death. The role of PP6 in regulating cell death requires its phosphatase activity and regulatory subunits. Further mechanistic studies show that PP6 modulates LUBAC-mediated M1-ubiquitination of RIPK1 and c-FLIP_L_ to promote RIPK1 activation and c-FLIP_L_ degradation. We also show that melanoma-associated PP6 inactivating mutants offer resistance to cell death due to the loss of sensitivity to TNFα. Thus, our study provides a potential mechanism by which melanoma-related PP6 inactivating mutations promote cancer progression.

## Introduction

Tumor necrosis factor α (TNFα) is a potent proinflammatory cytokine involved in a variety of human inflammatory and degenerative diseases [1–4]. Activation of TNFR1 by TNFα initiates the formation of sequential protein complexes that include signaling complex I and two alternative downstream execution complexes in cytosol, complex IIa and complex IIb [5, 6]. Complex I (also called TNF-RSC), formed rapidly in cells stimulated by TNFα, is associated with the intracellular death domain of TNFR1 which directly recruits DD-containing kinase RIPK1 and adaptor TRADD [7]. In complex I, TRADD recruits E3 ubiquitin ligases cIAP1/2 and linear ubiquitin assembly complex LUBAC, including HOIP, HOIL and Sharpin, which in turn modify RIPK1 by K63-ubiquitination and M1-ubiquitination, respectively [8–10]. The patterns of RIPK1 ubiquitination are important as they may serve as a code to decide distinct downstream cell fates [11]. In particular, the recruitment of LUBAC to complex I provides a critically important checkpoint that regulates the activation of RIPK1 by modulating the recruitment of key downstream regulators, including kinases, such as TBK1 and IKKα/β, and ubiquitin modulating proteins, such as A20, ABIN1 and NEMO [12–17]. Deficiency in any of these complex I components will promote the activation of RIPK1 to mediate RIPK1-dependent apoptosis (RDA) and necroptosis.

If the pattern of RIPK1 modification in complex I does not lead to the activation of its kinase activity, the cells will survive by promoting the activation of NF-κB pathway which mediates the expression of important pro-survival factors such as c-FLIP_L_, which is a direct inhibitor of caspase-8 [18, 19]. When the translational process is inhibited by cycloheximide (CHX), which blocks the expression of c-FLIP_L_, TNFα can drive the formation of a cytosolic complex (complex IIa) including RIPK1, FADD and caspase-8 and activation of caspase-8 to execute RIPK1-independent apoptosis (RIA) [20, 21]. Alternatively, the failure to inhibit the activation of RIPK1 kinase in complex I can promote the interaction of activated RIPK1 with FADD and caspase-8 to mediate RIPK1-dependent apoptosis (RDA); or when the activation of caspases are inhibited, activated RIPK1 can interact with RIPK3 and MLKL to form complex IIb (or necrosome) to mediate necroptosis [4, 22–24].

PP6, a member of the PPP family of Ser/Thr protein phosphatases, is highly evolutionarily conserved across species [25]. In mammalian cells, PP6 functions as a holoenzyme by interacting with its regulatory subunits, PPP6R1, PPP6R2, and PPP6R3. PP6 has been reported to participate in the regulation of cell cycle [26–28], DNA damage repair [29, 30] and tumorigenesis [31–35]. In particular, PP6 has been identified as a component of NF-κB interacting network in TNFα signaling pathway [36] and may be involved as a negative regulator of NF-κB pathway [37]. However, the target and mechanism by which PP6 may regulate the cell-fate decision upon TNFα stimulation are unclear.

Here we identify protein phosphatase PP6 as a previously unknown component of complex I. We show that the loss of PP6 protects cells from TNFα-mediated cell death. The role of PP6 in regulating cell death requires its phosphatase activity and regulatory subunits. We find that PP6 promotes TNFα-mediated RIPK1-independent cell death by regulating the proteasomal degradation of c-FLIP_L_. PP6 also promotes TNFα-mediated RIPK1-dependent cell death by regulating RIPK1 activation in complex I. Further mechanistic studies show that PP6 negatively modulates LUBAC-mediated M1-ubiquitination of RIPK1 and c-FLIP_L_ and thus promotes the kinase activation of RIPK1 and proteasome degradation of c-FLIP_L_. Finally, we show that the expression of melanoma-associated PP6 inactivating mutants offer resistance to TNFα. Thus, our study demonstrates that the regulation of LUBAC-mediated M1-ubiquitination of RIPK1 and c-FLIP_L_ by PP6 can serve as a checkpoint for TNFα-mediated cell death, which may provide a potential mechanism for promoting melanoma progression.

## Results

### PP6 is a previously unidentified component of complex I

**Fig. 1:**
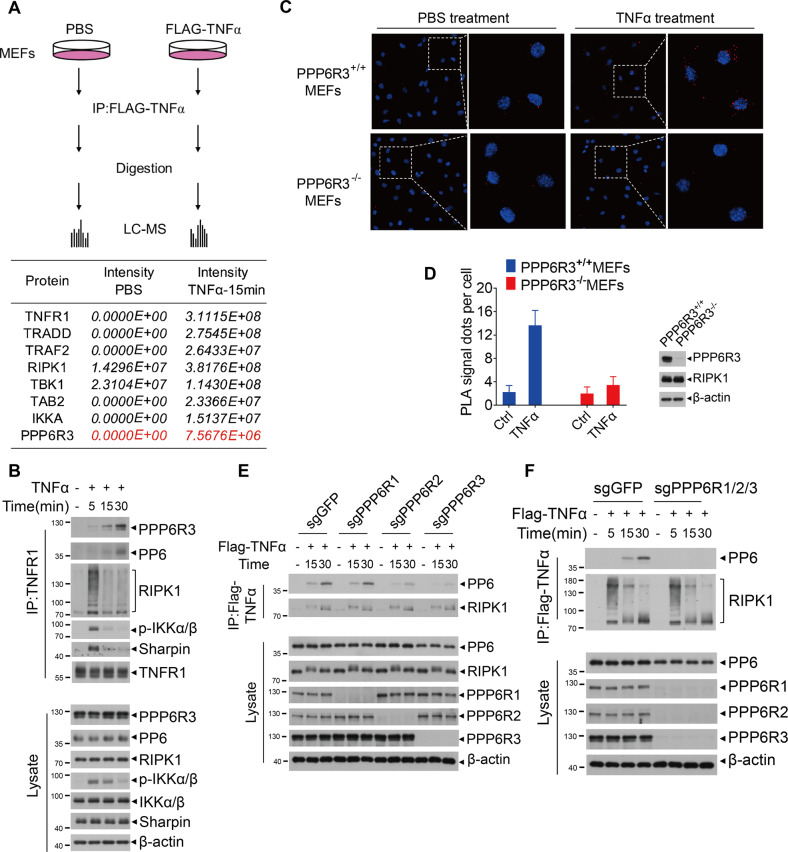
PP6 is a previously unidentified component of complex I. **A** MEFs were treated with Flag-TNFα or PBS for 15 min. Cells were lysed with NP-40 buffer and immunoprecipitated with anti-Flag beads. The immunocomplexes were analyzed and quantified by mass spectrometry. The protein abundance represented as intensity was quantified by the summed peptide intensities of all extracted Ion chromatograms (XICs). Table below showed the proteins enriched in Flag-TNFα treatment group. **B** MEFs were treated with TNFα for indicated periods of time. Cells were lysed with NP-40 buffer and immunoprecipitated with anti-TNFR1 antibody. The immunocomplexes and whole-cell lysates were analyzed by western blotting with indicated antibodies. **C** PPP6R3^+/+^ or PPP6R3^−/−^ MEFs were treated with PBS or TNFα for 30 min. In situ PLA assay was performed to detect the interaction between RIPK1 and PPP6R3. Red dots indicated the interaction signal of RIPK1 and PPP6R3. **D** Quantification analysis of the PLA signal dots of RIPK1 and PPP6R3. 20 cells were counted for each group. The panel on the right showed the knockout efficiency of PPP6R3. **E** PPP6R1, PPP6R2, or PPP6R3 knockout MEFs were treated with Flag-TNFα for indicated periods of time. Cells were lysed with NP-40 buffer and immunoprecipitated with anti-Flag beads. The immunocomplexes and whole-cell lysates were analyzed by western blotting with indicated antibodies. **F** WT or PPP6R1/2/3 triple-knockout MEFs were treated with Flag-TNFα for indicated periods of time. Cells were lysed with NP-40 buffer and immunoprecipitated with anti-Flag beads. The immunocomplexes and whole-cell lysates were analyzed by western blotting with indicated antibodies. Concentrations of reagents used: Flag-TNFα, 150 ng/ml; TNFα, 50 ng/ml.

To identify previously unknown regulators in complex I, we biochemically isolated native complex I from MEFs stimulated with Flag-TNFα by immunoprecipitation using anti-Flag affinity gel and analyzed the components of complex I by mass spectrometry analysis. PPP6R3, a regulatory subunit of PP6, was identified by mass spectrometry analysis in complex I after stimulation with Flag-TNFα for 15 min (Fig. 1A). Interestingly, in L929 cells stably expressing Flag-RIPK1, TNFα stimulation for 15 min also increased the binding of PPP6C, the catalytic subunit of PP6, with Flag-RIPK1 (Supplementary Fig. 1). PP6 was previously identified by TAP-MS as an interactor of IκBε and was involved in regulating NF-κB activity with PPP6R1 and PPP6R2 but not PPP6R3 [36, 38]. However, knockdown of IκBε showed no effect on necroptosis in previous genome-wide siRNA screening [39], which implicated the existance of unrevealed substrate of PP6 in complex I other than IκBε. PP6 is involved in a broad range of biological processes, a number of PP6 targets have been identified in different cellular signaling pathways [25]. These evidence prompted us to investigate if PP6 and its regulatory subunits might be components in complex I and the effect of PP6 on RIPK1 activation.

We confirmed the results from mass spectrometry analysis by immunoprecipitation. We found that in TNFα stimulated MEFs, PP6 and PPP6R3 were rapidly recruited into complex I together with RIPK1, IKKα/β, and Sharpin (Fig. 1B). We further utilized in situ proximity ligation assay (PLA) to detect the interaction of RIPK1 with PPP6R3 in cells. The interaction between RIPK1 and PPP6R3 was significantly enhanced upon TNFα stimulation as indicated by increased numbers of RIPK1-PPP6R3 signal dots in TNFα-treated MEFs compared to that of PBS-treated MEFs or TNFα-treated PPP6R3^−/−^ MEFs (Fig. 1C, D). These data suggest that PP6 and PPP6R3 are recruited to complex I upon TNFα stimulation.

SAPs domain-containing regulatory subunits of PP6 are important for the substrate specificity, subcellular localization and catalytic activity of PP6 holoenzyme [25, 38, 40]. In previous studies, knockdown of the associated regulatory subunits has shown equivalent effect to that of PP6 knockdown [38, 41–43]. To investigate the regulatory subunit involved in the recruitment of PP6 to complex I, PPP6R1, PPP6R2, or PPP6R3 were deleted individually by specific sgRNAs in MEFs. Interestingly, the recruitment of PP6 to complex I was unaffected by PPP6R1 knockout, partially reduced by PPP6R2 knockout and largely removed by PPP6R3 knockout (Fig. 1E), suggesting a partially redundant role of PPP6R2 and PPP6R3 in the recruitment of PP6 to complex I. We further developed a PPP6R1/2/3 triple-knockout MEF cell line and found that the recruitment of PP6 to complex I was totally abolished by PPP6R1/2/3 triple-knockout (Fig. 1F). ANKRDs (ankyrin repeat proteins) were also reported as regulatory subunits of PP6 that may contribute to its function and specificity [42]. None of the ANKRDs (Ankrd28, Ankrd44, Ankrd 52) was detected in our system, either by mass spectometry or by co-immunoprecipitation, suggesting that ANKRDs were dispensible for the recruitment of PP6 to complex I. Collectively, our data suggest that PP6 is a component of complex I, and the recruitment of PP6 to complex I depends on its regulatory subunits.

### PP6 promotes TNFα-mediated RIPK1-independent cell death by regulating c-FLIP_L_

**Fig. 2:**
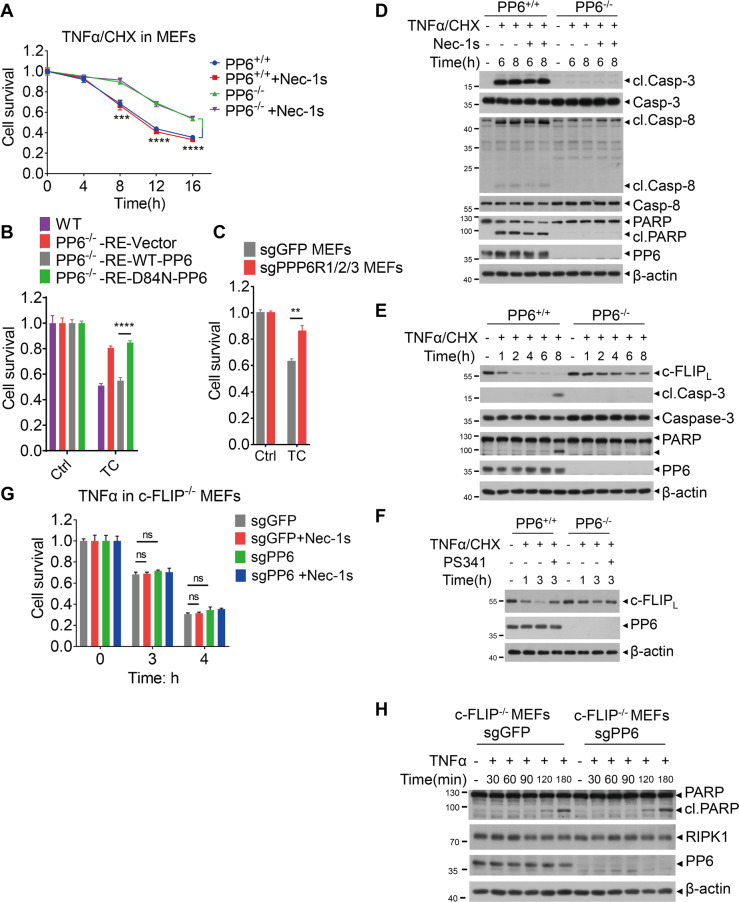
PP6 promotes TNFα-mediated RIPK1-independent cell death by regulating c-FLIP_L_. **A** PP6^+/+^ or PP6^−/−^ MEFs were pretreated with Nec-1s for 1 h and then treated with TNFα/CHX for indicated periods of time. **B** PP6^-/-^ MEFs were reconstituted with control vector, WT-PP6 or phosphatase-inactive D84N-PP6. Cells were then treated with TNFα/CHX for 10 h. **C** WT or PPP6R1/2/3 triple-knockout MEFs were treated with TNFα/CHX for 10 h. **D** PP6^+/+^ or PP6^−/−^ MEFs were pretreated with Nec-1s for 1 h and then treated with TNFα/CHX for indicated periods of time. Cells were lysed with RIPA buffer and analyzed by western blotting with indicated antibodies. **E** PP6^+/+^ or PP6^−/−^ MEFs were treated with TNFα/CHX for indicated periods of time. Cells were lysed with RIPA buffer and analyzed by western blotting with indicated antibodies. **F** PP6^+/+^ or PP6^−/−^ MEFs were pretreated with PS341 for 1 h and then treated with TNFα/CHX for indicated periods of time. Cells were lysed with RIPA buffer and analyzed by western blotting with indicated antibodies. **G** PP6 was removed in c-FLIP^−/−^ MEFs. Cells were pretreated with Nec-1s for 1 h and then treated with TNFα for indicated periods of time. **H** c-FLIP^−/−^ sgGFP or c-FLIP^−/−^ sgPP6 MEFs were treated with TNFα for indicated periods of time. Cells were lysed with RIPA buffer and analyzed by western blotting with indicated antibodies. Concentrations of reagents used: TNFα (T), 50 ng/ml; CHX (C), 2 μg/ml; Nec-1s, 10 μM; PS341, 300 nM. The cell death in (**A**–**C**, **G**) was measured by CellTiter-Glo assay. Data represent mean ± SD of three independent experiments (Student’s *t* test **P < 0.01; ***P < 0.001; ****P < 0.0001, ns not significant).

We next investigated the role of PP6 in TNFα-mediated cell death. PP6 was removed by specific sgRNA targeting PP6 in MEFs (Supplementary Fig. 2A). PP6 deficiency in MEFs reduced TNFα/CHX induced RIPK1-independent apoptosis (RIA), which was not affected by treatment cells with Nec-1s as expected (Fig. 2A). To examine if the catalytic activity of PP6 is required for PP6 to regulate cell death, we reconstituted PP6 knockout MEFs with control vector, WT-PP6 and phosphatase-inactive mutant D84N-PP6 (Supplementary Fig. 2B). The sensitivity of PP6 knockout MEFs to TNFα/CHX was restored by the expression of WT-PP6, but not by catalytically inactive D84N-PP6 (Fig. 2B). In addition, MEFs with PPP6R1/2/3 triple-knockout were also resistant to TNFα/CHX (Fig. 2C). Thus, the catalytic activity of PP6 and its regulatory subunits are involved in mediating RIPK1-independent apoptosis.

We next characterized the biochemical hallmarks of apoptosis in WT and PP6^−/−^ MEFs. We found that the cleavages of caspase-3, caspase-8 and PARP induced by TNFα/CHX were all inhibited by PP6 knockout (Fig. 2D). Since the cleavage of caspase-8 is an upstream event in apoptosis induced by TNFα/CHX, we examined the effect of PP6 on the levels of c-FLIP, which is an important negative regulator of caspase-8 activation [18, 19]. Interestingly, we found that the reduction in the levels of c-FLIP_L_ induced by TNFα/CHX was significantly decreased in PP6^−/−^ MEFs (Fig. 2E). The mRNA levels of *c-Flip* were unaffected by PP6 knockout (Supplementary Fig. 2C). Inhibition of proteasomal degradation by PS341 reduced the loss of c-FLIP_L_ in TNFα/CHX treated MEFs, supporting the role of proteasome in mediating its degradation [44]. The reduction in the levels of c-FLIP_L_ stimulated by TNFα/CHX was reduced by PP6 knockout (Fig. 2F). However, we found that PP6 knockout in c-FLIP^−/−^ MEFs could not provide resistance to TNFα induced apoptosis and PARP cleavage (Fig. 2G, H and Supplementary Fig. 2D). These data suggest that PP6 mediates the sensitivity to TNFα induced RIPK1-independent apoptosis by regulating the degradation of c-FLIP_L_.

### PP6 also promotes TNFα-mediated RIPK1-dependent cell death

**Fig. 3:**
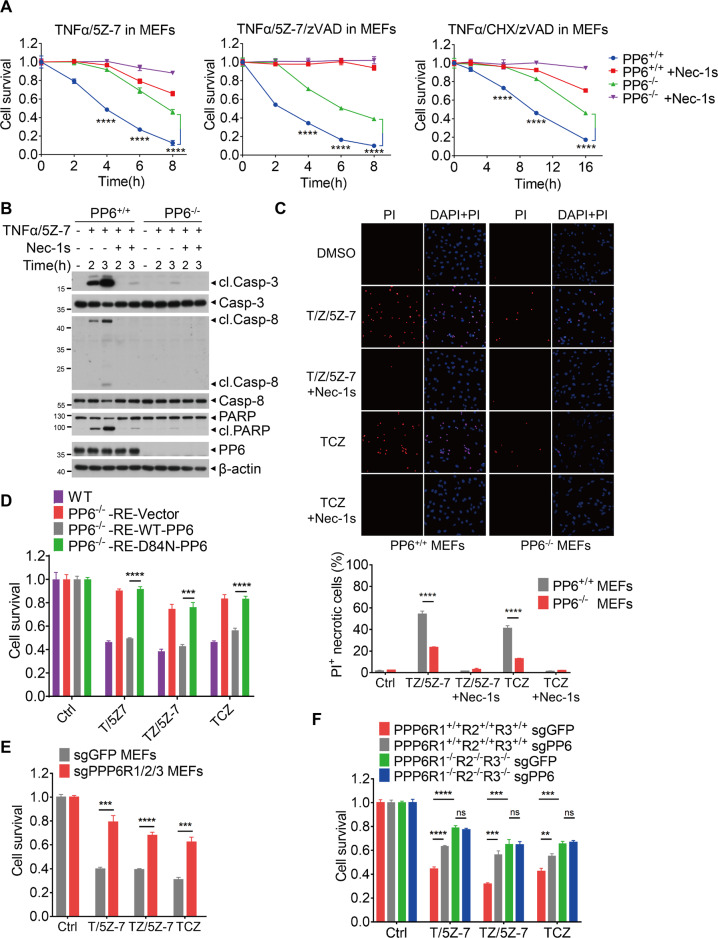
PP6 also promotes TNFα-mediated RIPK1-dependent cell death. **A** PP6^+/+^ or PP6^−/−^ MEFs were pretreated with Nec-1s for 1 h and then treated with TNFα/5Z-7, TNFα/zVAD/5Z-7 or TNFα/zVAD/CHX for indicated periods of time. **B** PP6^+/+^ or PP6^−/−^ MEFs were pretreated with Nec-1s for 1 h and then treated with TNFα/5Z-7 for indicated periods of time. Cells were lysed with RIPA buffer and analyzed by western blotting with indicated antibodies. **C** PP6^+/+^ or PP6^−/−^ MEFs were pretreated with Nec-1s for 1 h and then treated with TNFα/zVAD/5Z-7 for 3 h or TNFα/zVAD/CHX for 6 h. Cells were stained with DAPI and PI. The panel below showed the quantification result of PI positive cells. **D** PP6^−/−^ MEFs were reconstituted with control vector, WT-PP6 or phosphatase-inactive D84N-PP6. Cells were then treated with TNFα/5Z-7 and TNFα/zVAD/5Z-7 for 4 h or TNFα/zVAD/CHX for 8 h. **E** WT or PPP6R1/2/3 triple-knockout MEFs were treated with TNFα/5Z-7 and TNFα/zVAD/5Z-7 for 4 h or TNFα/zVAD/CHX for 8 h. **F** PP6 was removed in WT or PPP6R1/2/3 triple-knockout MEFs. Cells were treated with TNFα/5Z-7 and TNFα/zVAD/5Z-7 for 4 h or TNFα/zVAD/CHX for 8 h. Concentrations of reagents used: TNFα (T), 50 ng/ml; CHX (C), 2 μg/ml; Nec-1s, 10 μM; zVAD (Z), 50 μM; 5Z-7, 300 nM. The cell death in (**A**, **D**–**F**) was measured by CellTiter-Glo assay. Data represent mean ± SD of three independent experiments (Student’s *t* test **P < 0.01; ***P < 0.001; ****P < 0.0001, ns not significant).

PP6 deficiency also reduced TNFα/5Z-7 induced RIPK1-dependent apoptosis (RDA) and TNFα/zVAD/5Z-7 or TNFα/zVAD/CHX induced necroptosis (Fig. 3A). Treatment of cells with Nec-1s, a highly specific small molecule inhibitor of RIPK1 [45], was able to further protect PP6 deficient MEFs to RDA and necroptosis and thus, PP6 deficiency does not affect the protection by Nec-1s (Fig. 3A). The loss of PP6 also suppressed necroptosis in L929 cells induced by TNFα and TNFα/zVAD (Supplementary Fig. 3A). Biochemically, PP6 deficiency in MEFs blocked the cleavage of caspase-3, caspase-8 and PARP, induced by TNFα/5Z-7 (Fig. 3B). Moreover, necrotic cells as indicated by PI staining were dramatically reduced in TNFα/zVAD/5Z-7 or TNFα/zVAD/CHX treated PP6 knockout MEFs compared to that of WT MEFs (Fig. 3C). These results demonstrate that PP6 also promotes TNFα-mediated RIPK1-dependent cell death.

We also examined whether the phosphatase activity of PP6 was involved in regulating TNFα-mediated RIPK1-dependent cell death. We found that WT-PP6 reconstituted MEFs restored the sensitivity to TNFα-mediated cell death, while the cell viability in D84N-PP6 reconstituted MEFs was comparable to that of control PP6 knockout MEFs (Fig. 3D). A similar result was also found in WT-PP6 reconstituted PP6 knockout L929 cells (Supplementary Fig. 3B), suggesting that the phosphatase activity is required for PP6 to regulate TNFα-mediated cell death.

We next examined the role of PP6 regulatory subunits in TNFα-mediated RIPK1-dependent cell death. We found that the loss of PPP6R1 showed no effect on TNFα-mediated RIPK1-dependent cell death, while the loss of PPP6R2 or PPP6R3 reduced TNFα-mediated RIPK1-dependent cell death (Supplementary Fig. 3C). Additionally, PPP6R1/2/3 triple-knockout MEFs were more resistant to TNFα-induced cell death than that of PPP6R1, PPP6R2 or PPP6R3 single-knockout MEFs (Fig. 3E and Supplementary Fig. 3D). Since the protective effect of PPP6Rs knockout on TNFα induced cell death was highly correlated with the recruitment of PP6 to complex I (Fig. 1E, F), we hypothesized that knockout of all three PP6 regulatory subunits would abolish the role of PP6 in regulating cell death. To test this hypothesis, we knocked out PP6 in PPP6R1/2/3 triple-knockout MEFs (Supplementary Fig. 3E). We found that the deletion of PP6 in PPP6R1/2/3 triple-knockout cells showed no further protection against TNFα-induced cell death (Fig. 3F). Collectively, our data show that PP6 is a positive regulator of TNFα-mediated cell death, and the role of PP6 in regulating cell death requires its phosphatase activity and regulatory subunits.

### PP6 promotes the activation of RIPK1

**Fig. 4:**
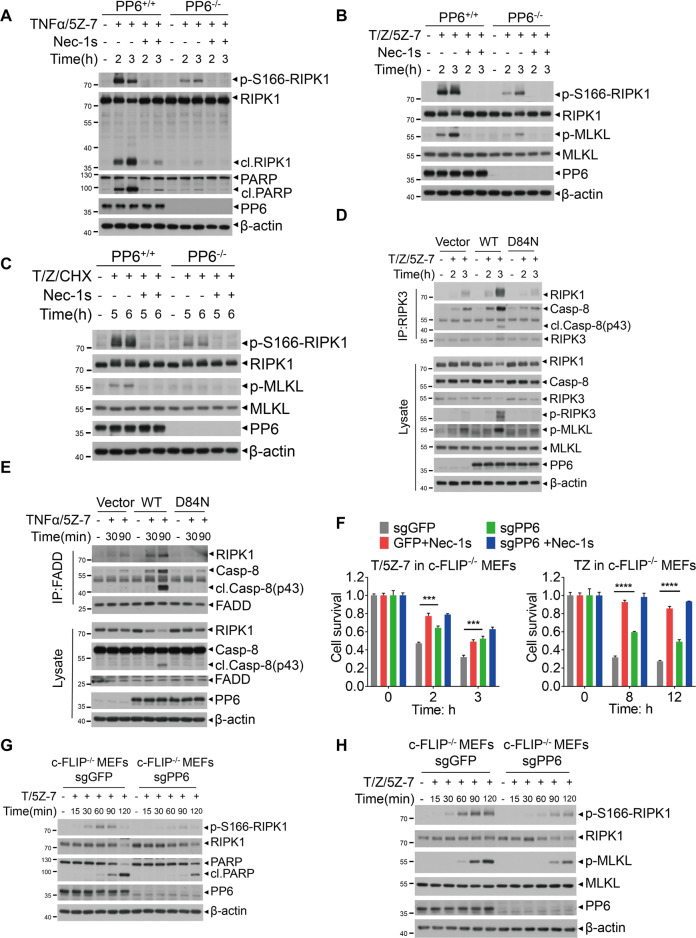
PP6 promotes the activation of RIPK1. **A**–**C** PP6^+/+^ or PP6^−/−^ MEFs were pretreated with Nec-1s for 1 h and then treated with TNFα/5Z-7, TNFα/zVAD/5Z-7, or TNFα/zVAD/CHX for indicated periods of time. Cells were lysed with RIPA buffer and analyzed by western blotting with indicated antibodies. **D**, **E** Control vector, WT-PP6 or D84N-PP6 reconstituted PP6^−/−^ MEFs were treated with TNFα/zVAD/5Z-7 or TNFα/5Z-7 for indicated periods of time. Cells were lysed with NP-40 buffer and immunoprecipitated with anti-RIPK3 or anti-FADD antibody. The immunocomplexes and whole-cell lysates were analyzed by western blotting with indicated antibodies. **F** c-FLIP^−/−^ sgGFP or c-FLIP^−/−^ sgPP6 MEFs were pretreated with Nec-1s for 1 h and then treated with TNFα/5Z-7 or TNFα/zVAD for indicated periods of time. **G**, **H** c-FLIP^−/−^ sgGFP or c-FLIP^−/−^ sgPP6 MEFs were treated with TNFα/5Z-7 or TNFα/zVAD/5Z-7 for indicated periods of time and then lysed with RIPA buffer. The lysates were analyzed by western blotting with indicated antibodies. Concentrations of reagents used: TNFα (T), 50 ng/ml; CHX (C), 2 μg/ml; Nec-1s, 10 μM; zVAD (Z), 50 μM; 5Z-7, 300 nM. The cell death in (**F**) was measured by CellTiter-Glo assay. Data represent mean ± SD of three independent experiments (Student’s *t* test ***P < 0.001; ****P < 0.0001).

Since the activation of RIPK1 kinase promotes both RIPK1-dependent apoptosis and necroptosis [4], we next examined the role of PP6 on the activation of RIPK1. We found that the activation of RIPK1, as indicated by its biomarker p-S166 [46, 47], in PP6^−/−^ MEFs stimulated by TNFα/5Z-7 was reduced compared to that of WT MEFs (Fig. 4A). The levels of p-S166 RIPK1 and p-S345 MLKL, the biomarker of necroptosis, were also reduced in PP6^−/−^ MEFs stimulated by TNFα/zVAD/5Z-7 or TNFα/zVAD/CHX, which induce necroptosis (Fig. 4B, C). The cleavages of RIPK1 and PARP, the downstream events in apoptosis, were also reduced in PP6^−/−^ MEFs treated with TNFα/5Z-7 compared to that of WT MEFs (Fig. 4A). Similarly, the activation of RIPK1 was also reduced in PPP6R1/2/3 triple-knockout MEFs (Supplementary Fig. 4A–C). Consistent with the involvement of PP6 in regulating the activation of RIPK1, reconstituting the expression of WT-PP6, but not catalytically inactive D84N mutant, restored the phosphorylation of T231/S232 RIPK3 and S345 MLKL and the formation of complex IIb in necroptosis (Fig. 4D) as well as the cleavage of caspase-8 and the formation of complex IIa in RDA (Fig. 4E). In addition, the formation of complex IIb induced by TNFα/zVAD/5Z-7 and complex IIa induced by TNFα/5Z-7 were decreased in PPP6R1/2/3 triple-knockout MEFs (Supplementary Fig. 4D, E). Thus, our data suggest that PP6 promotes the formation of both complex IIa and complex IIb in RDA and necroptosis, respectively.

Finally, we also examined the role of c-FLIP_L_ in PP6-regulated RDA and necroptosis. We found that knockout of PP6 could still increase the survival of c-FLIP^−/−^ MEFs induced by TNFα/5Z-7 or TNFα/zVAD, suggesting that c-FLIP_L_ is not essential for PP6 to mediate RDA or necroptosis (Fig. 4F). Furthermore, knockout of PP6 in c-FLIP^−/−^ MEFs could still reduce the levels of p-S166 RIPK1 and PARP cleavage induced by TNFα/5Z-7 (Fig. 4G) and also reduce the levels of p-S166 RIPK1 and p-S345 MLKL in c-FLIP^−/−^ MEFs treated with TNFα/zVAD/5Z-7 (Fig. 4H). Thus, unlike that of RIA, c-FLIP_L_ is not critical for PP6 to regulate RDA or necroptosis.

### HOIP is required for PP6 to regulate RIPK1 activation and c-FLIP_L_ degradation

**Fig. 5:**
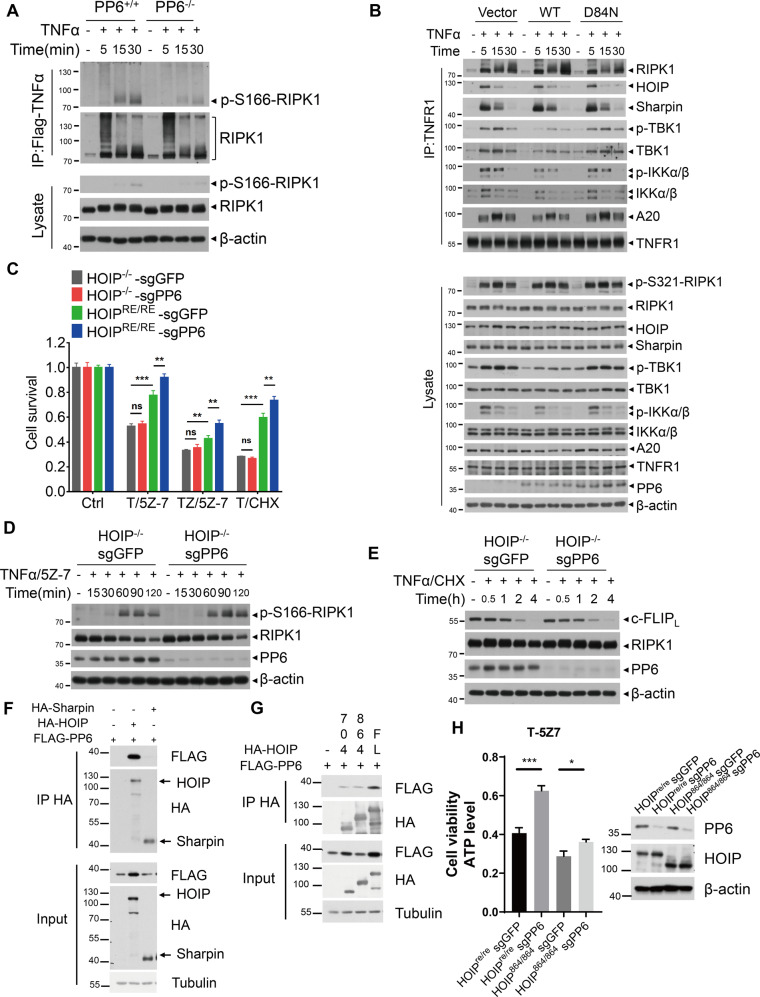
HOIP is required for PP6 to regulate RIPK1 activation and c-FLIP_L_ degradation. **A** PP6^+/+^ or PP6^−/−^ MEFs were treated with TNFα for indicated periods of time. Cells were lysed with NP-40 buffer and immunoprecipitated with anti-Flag beads. The immunocomplexes and whole-cell lysates were analyzed by western blotting with indicated antibodies. **B** Control vector, WT-PP6 or D84N-PP6 reconstituted PP6^−/−^ MEFs were treated with TNFα for indicated periods of time. Cells were lysed with NP-40 buffer and immunoprecipitated with anti-TNFR1 antibody. The immunocomplexes and whole-cell lysates were analyzed by western blotting with indicated antibodies. **C** PP6 was removed in HOIP^−/−^ MEFs and HOIP reconstituted MEFs. Cells were then treated with TNFα/5Z-7 and TNFα/zVAD/5Z-7 for 6 h or TNFα/CHX for 10 h. **D**, **E** HOIP^−/−^ sgGFP MEFs or HOIP^−/−^ sgPP6 MEFs were treated with TNFα/5Z-7 or TNFα/CHX for indicated periods of time. Cell were lysed with RIPA buffer and analyzed by western blotting with indicated antibodies. **F** FLAG-PP6 was co-expressed with HA-Sharpin or HA-HOIP in 293T cells for 24 h. Cells were lysed with NP-40 lysis buffer and immunoprecipitated with anti-HA beads. The immunocomplexes and whole-cell lysates were analyzed by western blotting with indicated antibodies. **G** FLAG-tagged PP6 was co-expressed with HA tagged WT or truncated (1-704 or 1-864) HOIP in 293T cells for 24 h. Cells were lysed with NP-40 lysis buffer and immunoprecipitated with anti-HA beads. The immunocomplexes and whole-cell lysates were analyzed by western blotting with indicated antibodies. **H** HOIP knockout MEFs reconstituted with HOIP(WT) or HOIP(1-864) truncation and with PP6 knockout were then treated with TNFα/5Z-7 for 8 h. Concentrations of reagents used: Flag-TNFα, 150 ng/ml; TNFα (T), 50 ng/ml; CHX (C), 2 μg/ml; zVAD (Z), 50 μM; 5Z-7, 300 nM. The cell death in (**C**, **H**) was measured by CellTiter-Glo assay. Data represent mean ± SD of three independent experiments (Student’s *t* test *P < 0.05; **P < 0.01; ***P < 0.001, ns not significant).

To further investigate the mechanism of PP6 in regulating TNFα-mediated cell death, we next examined the role of PP6 in mediating the activation of RIPK1 in complex I. We found that the activation of RIPK1 in complex I induced by TNFα or TNFα/5Z-7 was reduced by PP6 knockout (Fig. 5A, Supplementary Fig. 5A). To examine the mechanism by which PP6 deficiency protects against RIPK1 activation, we next characterized the recruitment of A20, TBK1 and IKKα/β in complex I, which are all known to negatively regulate the activation of RIPK1 [13, 48, 49]. Interestingly, we found that the recruitment of A20, TBK1 and IKKα/β as well as the phosphorylation of TBK1 and IKKα/β in complex I were all increased in PP6^−/−^ MEFs which was restored by complementation with WT-PP6, but not by D84N-PP6 mutant (Fig. 5B). In addition, triple-knockout of PPP6R1/2/3 also increased the recruitment of TBK1, IKKα/β, and A20 in complex I (Supplementary Fig. 5B). Thus, PP6 deficiency might reduce the activation of RIPK1 by promoting the recruitment and activation of multiple inhibitors of RIPK1, including A20, TBK1, and IKKα/β.

Since the recruitment of TBK1, IKKα/β and A20 to complex I is mediated by LUBAC [12–15], these data suggest that PP6 may regulate LUBAC. Indeed, although the recruitment of RIPK1, HOIP and Sharpin to complex I was unaffected by PP6 deficiency (Fig. 5B), we found that PP6 deficiency failed to protect HOIP^−/−^ MEFs from TNFα-mediated cell death, while the PP6 deficiency significantly reduced TNFα-mediated cell death in HOIP reconstituted MEFs (Fig. 5C and Supplementary Fig. 5C). Consistently, PP6 knockout could not protect the activation of RIPK1 in HOIP^−/−^ MEFs treated with TNFα/5Z-7 to induce RDA or with TNFα/zVAD/5Z-7 to induce necroptosis (Fig. 5D and Supplementary Fig. 5D).

cIAP1/2 are involved in the recruitment of LUBAC to complex I [9]. Consistently, we found that PP6 knockout failed to protect against TNFα-mediated cell death in cIAP1/2 double-knockout MEFs (Supplementary Fig. 5E). Moreover, inhibition of cIAP1/2 by Smac mimetic SM-164 [50] also impaired the ability of PP6 deficiency to protect against TNFα-mediated cell death (Supplementary Fig. 5F).

We also investigated the role of LUBAC on c-FLIP_L_ degradation in HOIP^−/−^ MEFs. Interestingly, unlike that of WT MEFs (Fig. 2E), PP6 deficiency in HOIP^−/−^ MEFs had no effect on c-FLIP_L_ degradation (Fig. 5E). Similarly, the effect of PP6 deficiency to block RIPK1 activation as well as c-FLIP_L_ degradation was impaired in cells treated with cIAP1/2 inhibitor SM-164 (Supplementary Fig. 5G–I). Thus, these results demonstrate that LUBAC is critical for PP6 to regulate RIPK1 activation and c-FLIP_L_ degradation.

To further dissect the molecular mechanism underlying the recruitment of PP6, we checked the interaction between LUBAC and PP6, and found that HOIP but not Sharpin can directly bind with PP6 (Fig. 5F). Furthermore, the catalytic activity of HOIP was required for the binding with PP6. As shown in Fig. 5G, deletion of RBR-LDD (705A.A.−1066A.A) or R-LDD (CBR, 865A.A.−1066A.A.) domains, which encode the catalytic core of HOIP [51] (Supplementary Fig. 5J), resulted in the loss of interaction with PP6. Furthermore, reconstitution of WT HOIP, but not ΔCBR HOIP, was able to restore the effect of PP6 in regulating cell death (Fig. 5H). These data suggest that HOIP is involved in the recruitment of PP6 to complex I associated with TNFR1.

### PP6 negatively modulates LUBAC-mediated M1-ubiquitination of RIPK1 and c-FLIP_L_

**Fig. 6:**
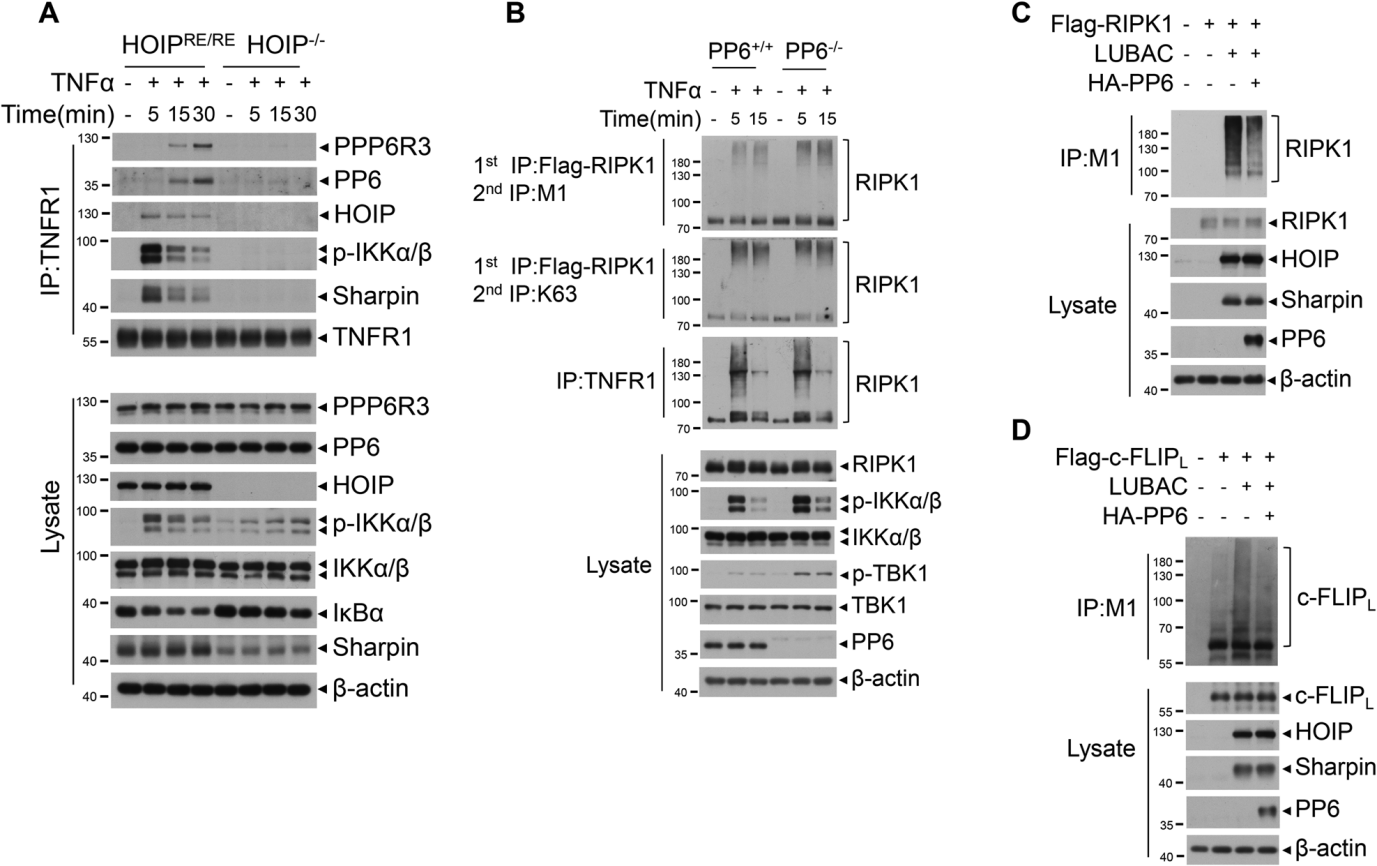
PP6 negatively modulates LUBAC-mediated M1-ubiquitination of RIPK1 and c-FLIP_L_. **A** HOIP^−/−^ or HOIP reconstituted MEFs were treated with TNFα for indicated periods time. Cells were lysed with NP-40 buffer and immunoprecipitated with anti-TNFR1 antibody. The immunocomplexes and whole-cell lysates were analyzed by western blotting with indicated antibodies. **B** PP6^+/+^ and PP6^−/−^ L929 cells with Flag-RIPK1 stable expression were treated with TNFα for indicated periods of time. Cells were lysed with NP-40 buffer and immunoprecipitated with anti-Flag beads. The isolated complex I was eluted with urea lysis buffer. The eluted complex I was further immunoprecipitated with chain specific M1 or K63 ubiquitin antibody. The immunocomplexes and whole-cell lysates were analyzed by western blotting with indicated antibodies. **C** Flag-RIPK1 was co-expressed with LUBAC (HA-HOIP/Myc-HOIL/Flag-Sharpin) or HA-PP6 in 293T cells for 20 h. Cells were lysed with urea lysis buffer and immunoprecipitated with chain specific M1 ubiquitin antibody. The immunocomplexes and whole-cell lysates were analyzed by western blotting with indicated antibodies. **D** Flag-tagged c-FLIP_L_ was co-expressed with LUBAC (HA-HOIP/Myc-HOIL/Flag-Sharpin) or HA-PP6 in 293T cells for 20 h. Cells were treated with PS341 8 h after transfection. Cells were lysed with urea lysis buffer and immunoprecipitated with chain specific M1 ubiquitin antibody. The immunocomplexes and whole-cell lysates were analyzed by western blotting with indicated antibodies. Concentrations of reagents used: TNFα (T), 50 ng/ml; PS341, 100 nM.

We next examined the recruitment of PP6 and PPP6R3 to complex I in HOIP^-/-^ MEFs. We found that both PP6 and PPP6R3 failed to be recruited into complex I in HOIP^−/−^ MEFs stimulated by TNFα (Fig. 6A). In addition, we found that M1-ubiquitination of RIPK1 was increased, while that of K63 ubiquitination was unaffected, in PP6^−/−^ MEFs stimulated by TNFα (Fig. 6B). Moreover, the M1-ubiquitination of RIPK1 mediated by LUBAC was reduced by co-expression of PP6 in 293T overexpressed cells (Fig. 6C). The catalytic activity of PP6 was required for the regulation of LUBAC ligase activity, as overexpression of inactive D84N mutant showed attenuated ability to suppress RIPK1 linear ubiquitination (Supplementary Fig. 6). Since the recruitment of LUBAC to complex I was not affected by PP6 knockout (Fig. 5B), these data strongly suggest that PP6 can promote RIPK1 activation by negatively regulating LUBAC-mediated M1-ubiquitination of RIPK1.

c-FLIP_L_ was recently reported to be modified with M1-ubiquitination by LUBAC in TNFα induced apoptosis [52]. We found that the M1-ubiquitination of c-FLIP_L_ mediated by LUBAC was reduced by co-expression of PP6 in 293T overexpressed cells (Fig. 6D), suggesting that PP6 can promote c-FLIP_L_ degradation by reducing LUBAC-mediated M1-ubiquitination of c-FLIP_L_. Taken together, our data indicate that PP6 can promote RIPK1 activation and c-FLIP_L_ degradation by reducing the LUBAC activity in TNFα signaling.

### Melanoma-associated PP6 inactivating mutations reduce the sensitivity to TNFα

**Fig. 7:**
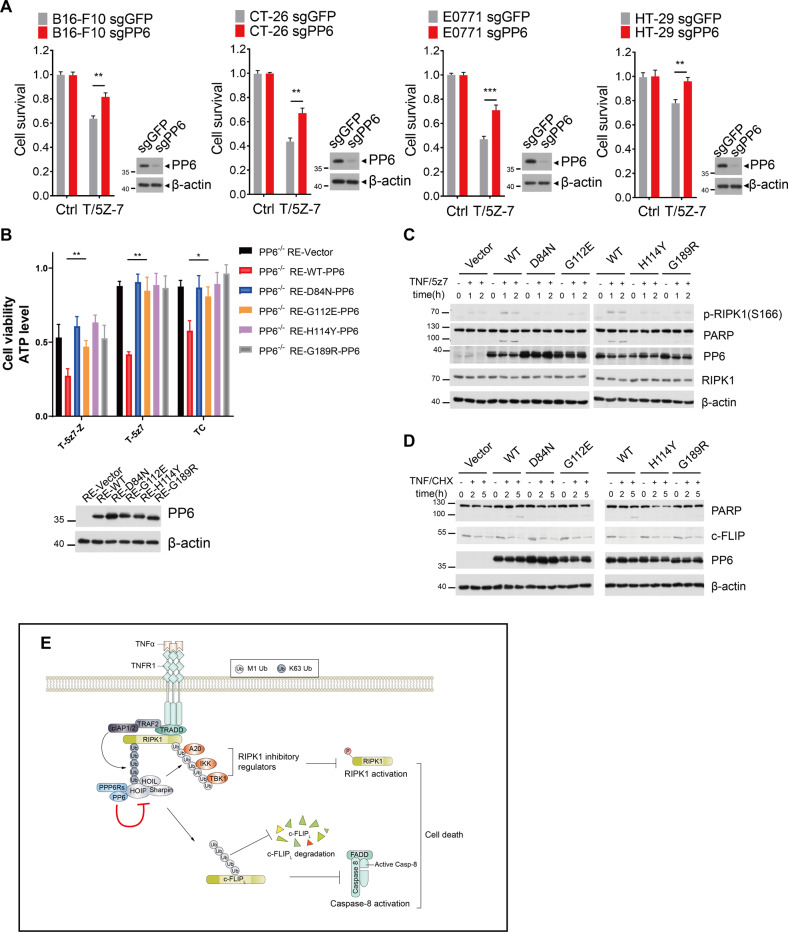
Melanoma-associated PP6 inactivating mutations reduce the sensitivity to TNFα. **A** PP6 was removed by specific sgRNA in B16-F10 (murine melanoma cell), CT-26 (murine colorectal carcinoma cells), E0771 (murine breast adenocarcinoma cells) and HT-29 (human colorectal adenocarcinoma cell) tumor cells. Cells were then treated with TNFα/5Z-7 for 6 h. **B** PP6^−/−^ MEFs were reconstituted with control vector, WT-PP6, D84N-PP6, or melanoma-associated G112E-PP6, H114Y-PP6, and G189R-PP6. Cells were then treated with TNFα/zVAD/5Z-7 and TNFα/5Z-7 for 4 h or TNFα/CHX for 10 h. The panel on the right showed the efficiency of PP6 reconstitution. **C**, **D** Control vector, WT-PP6, D84N-PP6, or melanoma-associated G112E-PP6, H114Y-PP6, and G189R-PP6 reconstituted PP6−/− MEFs were treated with TNFα/5Z-7 or TNFα/CHX for indicated periods of time. Cells were lysed with RIPA buffer and analyzed by western blotting with indicated antibodies. **E** A schematic model shows that PP6 negatively modulates LUBAC-mediated M1-ubiquitination of RIPK1 and c-FLIP_L_ to promote TNFα-mediated cell death. Concentrations of reagents used: TNFα (T), 50 ng/ml; CHX (C), 2 μg/ml; zVAD (Z), 50 μM; 5Z-7, 300 nM. The cell death in (**A**, **B**) was measured by CellTiter-Glo assay. Data represent mean ± SD of three independent experiments (Student’s *t* test *P < 0.05; **P < 0.01; ***P < 0.001).

PP6 was identified as a risk gene of melanoma as it is mutated in nearly 9% of melanoma characterized [31, 53]. At least some melanoma-associated PP6 mutations were known to lead to reduction or loss of the phosphatase activity [54]. We found that knockout of PP6 in various cancer cell lines including B16-F10 melanoma cells reduced the sensitivity to TNFα (Fig. 7A). Since our data showed that phosphatase-inactive D84N-PP6 mutant cells were resistant to TNFα-mediated cell death (Figs. 2B, 3D), we next examined whether melanoma-associated PP6 inactivating mutations would affect TNFα-mediated cell death. Melanoma-associated inactivated mutations of PP6 were reconstituted to PP6^−/−^ MEFs. We found that while WT-PP6 reconstituted PP6^−/−^ MEFs were highly sensitive to TNFα-mediated cell death; PP6^−/−^ MEFs reconstituted with phosphatase-inactive D84N-PP6 or melanoma-associated G112E, H114Y, and G189R mutants remained resistant to TNFα induced cell death (Fig. 7B). Consistently, the activation of RIPK1 and the degradation of c-FLIP_L_ was also reduced by phosphatase dead D84N mutant and melanoma-associated G112E, H114Y, and G189R mutants (Fig. 7C, D). Thus, our data demonstrate that melanoma-associated PP6 mutations mediates the resistance to TNFα.

## Discussion

In this study, we identify PP6 as a positive regulator of both RIPK1-dependent and independent cell death mediated by TNFα. We show that PP6 is recruited to TNFR1-complex I mediated by HOIP and promotes TNFα-mediated cell death by promoting RIPK1 activation and c-FLIP_L_ degradation (Fig. 7E). c-FLIP_L_ is required for PP6 to regulate RIA, but not RDA or necroptosis. Because RIPK1 kinase is not involved in RIA, PP6-mediated regulation of c-FLIP_L_ plays a dominant role in RIA. However, PP6-mediated regulation of both RIPK1 and c-FLIP_L_ participates in RDA and necroptosis. Our data suggest that PP6 and its regulatory subunits PPP6R3 and PPP6R2 are involved in mediating TNFα signaling, while PPP6R1 is not involved or plays a redundant role in mediating TNFα signaling pathway. The N-terminal SAPS domains of PP6 regulatory subunits involved in binding with PP6 are highly conserved, while the C-terminal protein sequences of PPP6Rs are less conserved which may enable the binding with specific substrates of PP6 holoenzymes. PPP6R3 shares a higher degree of homology with PPP6R2 than PPP6R1, which may explain why PPP6R2 and PPP6R3, but not PPP6R1, can regulate TNFα signaling.

LUBAC functions as a critical modulator of TNFα signaling in the activation of NF-κB and prevention of cell death. We show that PP6 promotes RIPK1 activation and c-FLIP_L_ degradation by restraining LUBAC-mediated M1-ubiquitination of these substrates which in turn affects the recruitment of RIPK1 regulators such as A20, TBK1 and IKK. Of note, the expression of PP6 can reduce the M1-ubiquitination of RIPK1 and c-FLIP_L_ in 293T overexpressed system, which suggests that PP6 may directly regulate LUBAC activity. Previous studies have shown that the activity of LUBAC can be regulated by the phosphorylation of certain components within the complex [55–57]. Accordingly, further mechanistic studies need to be performed to determine the substrate specificity of PP6 in LUBAC complex. Since PP6 is a member of PPP family of protein Ser/Thr phosphatases whose specificities may be conveyed by their associated regulatory subunits [58], future efforts will be needed to map the interactions of different LUBAC components with PP6 and its three regulatory subunits.

Recently, several CRISPR/Cas9-mediated genetic screening studies have revealed that the loss of certain pro-survival components such as HOIP, HOIL, TBK1, IKKβ or c-FLIP in TNFα signaling would sensitize tumor cells to CD8^+^ T cell- and NK cell-mediated killing [59–62]; in contrast, the loss of TNFR1, caspase-8 or FADD protects tumor cells against CD8^+^ T cell and NK cell cytotoxicity [62–64]. These screens indicated that TNFα-mediated tumor cell killing induced by cytotoxic T lymphocytes and NK cells plays a critical role in antitumor immunity, and tumor cells may take advantage of pro-survival TNFα signaling to avoid TNFα-mediated cell death during immune surveillance. We show that PP6 deficiency offers resistance to TNFα-mediated cell death due to increased activation of pro-survival TBK1 and IKKβ in complex I and enhanced stability of anti-cell death protein c-FLIP_L_. Moreover, we find that PP6 inactivating mutations reduced the sensitivity to TNFα. Our data suggest that PP6 deficiency or phosphatase inactivation may confer a survival advantage to tumor cells in response to TNFα-mediated killing in tumor microenvironment and thereby may allow immune evasion to promote tumorigenesis.

PP6 has been found to be frequently mutated in melanoma and therefore known to be a risk factor of melanoma [31, 53]. Keratinocyte-specific PP6 deficient mice show increased susceptibility to UV and DMBA induced skin carcinogenesis [32, 35]. Thus, PP6 is a critical tumor suppressor in skin tumorigenesis. Of note, LUBAC plays an important role in the resistance to CD8^+^ T cell- and NK cell-mediated killing in murine B16-F10 and human D10 melanoma cells [59–61]. Our data suggest that PP6 might be a negative modulator of LUBAC that maintains the sensitivity to TNFα by negatively modulating M1-ubiquitination of RIPK1 and c-FLIP_L_. We further show that PP6 deficient tumor cells and cells expressing melanoma-associated PP6 mutants are less sensitive to TNFα treatment. Therefore, the discovery for the role of PP6 in modulating TNFα-mediated cell death provides new insights into the mechanism that promotes melanoma progression. Our study also suggests that inhibition of LUBAC activity in tumors harboring PP6 mutations may provide therapeutical benefits.

## Materials and methods

### Antibodies and reagents

The following antibodies and reagents were used in this study: PP6 (15852-1-AP), PPP6R1 (17819-1-AP), PPP6R3 (16944-1-AP) and Sharpin (14626-1-AP) were purchased from Proteintech Group. PPP6R2 (sc-376238) and FADD (sc-6036) were purchased from Santa Cruz. Caspase-8 (4927), Cl-Caspase-8 (8592), Caspase-3 (9662), Cl-Caspase-3 (9661), c-FLIP (56343), PARP (9532), IKKα (2682), IKKβ (2678), p-IKKα/β (2697), RIPK1 (3493), p-S166-RIPK1 (31122), p-T231/S232-RIPK3 (57220), TBK1 (3504), p-S172-TBK1 (5483) and A20 (5630) were purchased from Cell Signaling Technology. MLKL (ab183770), p-S345-MLKL (ab196436), and FADD (ab124812) were purchased from Abcam. TNFR1 (AF-425-PB) and pan-cIAP (MAB3400) were purchased from R&D Systems. β-actin (I10813) was purchased from TransGen. Human recombinant TNFα (C008) was purchased from Novoprotein. 5Z-7 (O9890) and CHX (C7698) were purchased from Sigma. PS341 and (HY-10227) and zVAD.fmk (HY-16658) were purchased from MedChemExpress. M1 and K63 ubiquitin antibodys were gifts from Dr. Vishva Dixit of Genentech [65].

### Cell culture

MEFs, L929 cells and HEK293T cells were cultured in DMEM (Gibco) supplemented with 10% (v/v) FBS (Gibco) and 1% (v/v) streptomycin (100 μg/ml)/penicillin (100 units/mL). Cells were cultured at 37 °C with 5% CO2.

### Lentivirus-mediated CRISPR knockout and reconstitution

Lentivirus were produced in HEK293T cells by the transfection of sgRNA plasmids or re-expression plasmids with packaging plasmids. Lentivirus was collected 48 h post-transfection. MEFs and L929 cells were infected with lentivirus containing CRISPR-sgRNAs targeting PP6 or PPP6Rs. Knockout cells were then selected by puromycin. PP6 and PPP6Rs sgRNA sequences are listed below:

sgPP6: 5′-AAATACGGCGTCGTGGTTCT-3′;

sgPPP6R1-1: 5′-GCGATCAAGCCGGGGACGCA-3′;

sgPPP6R1-2: 5′-CAACGCCGCTGCCTTGACGC-3′;

sgPPP6R1-3: 5′-GGATCATCTGCTCGCGGCTT-3′;

sgPPP6R2-1: 5′-GCCATGGCTCGATGCCACGA-3′;

sgPPP6R2-2: 5′-GATCAGCGACAGACTAGGCG-3′;

sgPPP6R2-3: 5′-GTCCAGGAGCTTGTCAACGT-3′;

sgPPP6R3-1: 5′-GAACAGCATTGGCGTCATAT-3′;

sgPPP6R3-2: 5′-TACAGTTAGCTATCCTCGTA-3′;

sgPPP6R3-3: 5′-GTTACATGGGACACCTTACG-3′;

For reconstitution of PP6 in PP6^−/−^ cells, sgRNA-resistant PP6 plasmids were designed by synonymous mutating the sgRNA sequence targeting PP6. The reverse primer sequence used for the construction of sgRNA-resistant PP6 expression plasmid is listed below:

sgPP6-resistant: 5′-TCACAAAAAGTACGGCGTCGTGGTTCTGGGAGGAA-3′.

### Cell viability assays

Cell viability was determined by measuring the ATP luminescence using CellTiter-Glo assay (Promega) according to the manufacturer’s protocol. For PI staining assay, PI and DAPI were added to cells for 10 min. Pictures were taken by high-content analysis system (PerkinElmer).

### In situ PLA assay

In situ PLA assay was performed as previously described [66]. Briefly, cells were fixed with 4% paraformaldehyde for 15 min and then permeabilized in 0.1% Triton X-100 for 15 min. Cells were treated with blocking buffer and then incubated with anti-RIPK1 (homemade, 1:2000 dilution) and anti-PPP6R3 (16944-1-AP, Proteintech Group, 1:2000 dilution) for 12 h at 4 °C. Cells were further incubated with secondary antibodies and followed by the ligation and amplification processes. PLA signal dots were finally taken by fluorescence microscope and counted using ImageJ.

### Immunoblots and immunoprecipitation

For immunoblots, cells were lysed in RIPA buffer containing 10 mM Tris-HCl, 150 mM NaCl, 0.1% SDS, 1% Triton-X-100, 1 mM EDTA, 1 mM EGTA, 5 mM sodium pyrophosphate, 10 mM β-glycerophosphate, 5 mM NaF, 1 mM Na_3_VO_4_, 0.5% sodium deoxycholate and 10%(v/v) glycerol (PH = 7.4). 1× Protease inhibitor cocktail (B14001, Biomake), 1 mM PMSF and 5 mM N-ethylmaleimide (E3876, Sigma) were freshly added to the lysis buffer prior to use. The protein concentrations of collected supernatants were determined by the Pierce BCA Protein Assay Kit (23225, ThermoFisher) and normalized to the same concentration. Lysates were then diluted in 5x loading buffer at 95 °C for 10 min and then analyzed by SDS-PAGE.

For immunoprecipitation of complex I or complex II, cells were lysed in NP-40 buffer containing 50 mM Tris-HCl, 150 mM NaCl, 1% NP-40, 1 mM EDTA, 1 mM EGTA, 2.5 mM sodium pyrophosphate, 10 mM β-glycerophosphate, 5 mM NaF, 1 mM Na_3_VO_4_ and 10%(v/v) glycerol (PH = 7.4). 1× Protease inhibitor cocktail, 1 mM PMSF and 5 mM N-ethylmaleimide were freshly added to the lysis buffer prior to use. The supernatants were incubated with corresponding antibodies overnight at 4 °C and then incubated with Protein G agarose (101242, ThermoFisher) for 4 h. For immunoprecipitation of Flag-TNFα, the cell lysates were incubated with anti-Flag beads (A2220, Sigma) overnight at 4 °C.

For immunoprecipitation of K63 or M1 ubiquitin in complex I, the complex I was firstly isolated by anti-Flag beads in TNFα stimulated Flag-RIPK1 stably expressed L929 cells. The isolated complex I was further eluted with urea lysis buffer containing 6 M urea, 100 mM Tris-HCl, 150 mM NaCl, 5 mM EDTA, 1.5 mM MgCl_2_, 1% Triton X-100 and 1x Protease inhibitor cocktail (PH = 8.0) at 4 °C for 2 h. The eluted complex I was incubated with chain specific K63 ubiquitin antibody (1.5 μg for each sample, diluted with lysis buffer without urea to bring the final urea concentration to 3 M) or M1 ubiquitin antibody (3.0 μg for each sample) overnight at 4 °C followed by the incubation of Protease A agarose (20333, Pierce) for 4 h.

For immunoprecipitation of K63 or M1 ubiquitin in 293T overexpressed cells, cells were lysed in urea lysis buffer. The lysates were flash frozen in liquid nitrogen. The supernatants were incubated with chain specific K63 ubiquitin antibody (1.5 μg for each sample, diluted with lysis buffer without urea to bring the final urea concentration to 3 M) or M1 ubiquitin antibody (3.0 μg for each sample) overnight at 4 °C followed by the incubation of Protease A agarose (20333, Pierce) for 4 h. All immunocomplexes were eluted with 2× loading buffer at 95 °C for 10 min and then analyzed by SDS-PAGE.

### Mass spectrometry

Cells were treated with Flag-TNFα for 15 min and then lysed with NP-40 buffer. After co-IP against Flag-TNFα, the binding proteins of TNFR1 were trypsin digested on beads. The peptides were analyzed on Q Exactive HF Hybrid Quadrupole-Orbitrap Mass Spectrometer (Thermo Scientific). Protein identification and quantification was performed by MaxQuant [67]. The tandem mass spectra were searched against UniProt mouse protein database. The precursor and fragment mass tolerance were set as 20 ppm. The FDR at peptide spectrum match levels and protein levels were controlled below 1%. The unique peptides plus razor peptides were included for quantification.

### Statistics

All cell viability results were processed using GraphPad Prism 7. Results shown depict the mean (±s.e.m.) of at least three independent biological replicates. P values were calculated by two-tailed Student’s *t* test (*P < 0.05; **P < 0.01; ***P < 0.001; ****P < 0.0001). Each individual experiment was repeated at least 3 times.

### Reporting summary

Further information on research design is available in the Nature Research Reporting Summary linked to this article.

## Supplementary information

Supplementary Figures

reporting summary

## Data Availability

The mass spectrometry proteomics data have been deposited to the ProteomeXchange Consortium (http://proteomecentral.proteomexchange.org) via the iProX partner repository [68] with the dataset identifier PXD030377.
