# Supplementary material for: PP6 negatively modulates LUBAC-mediated M1-ubiquitination of RIPK1 and c-FLIPL to promote TNFα-mediated cell death
Source: Cell Death Dis. 2022 Sep 7;13(9):773. doi: 10.1038/s41419-022-05206-9 (PMC9452587; doi:10.1038/s41419-022-05206-9)
Supplement: Supplementary file 1 — Supplementary Figures [file 41419_2022_5206_MOESM1_ESM.pdf]

PP6 negatively modulates LUBAC-mediated M1-ubiquitination of RIPK1 and c-FLIP$_L$ to promote TNFα-mediated cell death

Guowei Wu[1,2,3], Dekang Li[1,2,3], Wei Liang[1,2], Weimin Sun[1,2], Xingxing Xie[1,2], Yilun Tong[1,2], Bing Shan[1], Mengmeng Zhang[1], Xiaojuan Lu[1], Junying Yuan[1]* and Ying Li[1]*.

[1]Interdisciplinary Research Center on Biology and Chemistry, Shanghai Institute of Organic Chemistry, Chinese Academy of Sciences, 100 Haike Road, PuDong District, Shanghai 201210, China.

[2]University of Chinese Academy of Sciences, Beijing 100049, China.

[3]These authors contributed equally.

*Corresponding authors.

Contact author: Junying Yuan. Email: junying_yuan@sioc.ac.cn

Ying Li, Email: liying@sioc.ac.cn

Supplementary Figures

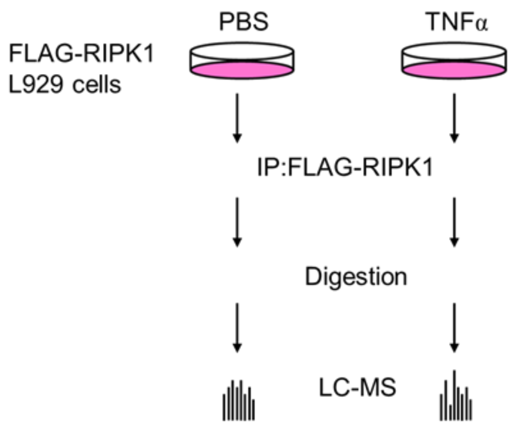

Protein	Intensity PBS	Intensity TNFα-15min
RIPK1	*3.4727E+10*	*4.5076E+10*
TNFR1	*0.0000E+00*	*1.9282E+08*
TRADD	*0.0000E+00*	*2.2172E+08*
PPP6C	*8.8995E+06*	*2.6912E+07*

Supplementary Fig. 1 PP6 is a previously unidentified complex I component. Flag-RIPK1 stably expressed L929 cells were treated with TNFα or PBS for 15 min. Cells were lysed with NP-40 buffer and immunoprecipitated with anti-Flag beads. Flag-RIPK1 immunocomplexes were analyzed and quantified by mass spectrometry. The protein abundance represented as intensity were quantified by the summed peptide intensities of all extracted Ion chromatograms (XICs). Table below showed the proteins enrich in TNFα treatment group. Concentration of reagent used: TNFα, 50 ng/ml.

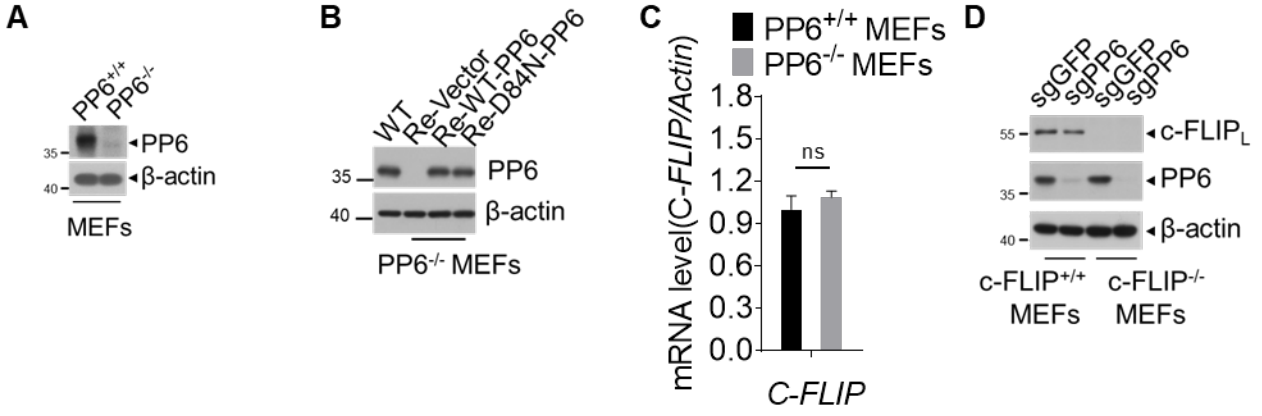

Supplementary Fig. 2 PP6 promotes TNFα-mediated RIPK1-independent cell death by regulating c-FLIP$_L$. A MEFs were transfected with lentivirus containing sgRNA targeting PP6 to knockout PP6. PP6 knockout efficiency was confirmed by western blotting. **B** PP6$^{-/-}$ MEFs were reconstituted with control vector, WT-PP6 or phosphatase inactive D84N-PP6. PP6 reconstitution efficiency was confirmed by western blotting. **C** PP6$^{+/+}$ or PP6$^{-/-}$ MEFs were lysed with RNAiso reagent and the mRNA was extracted and reverse-transcribed into cDNA. The basal mRNA level of *c-FLIP* was determined by quantitative real-time PCR with specific c-FLIP primers. *Actin* was used as loading control. **D** PP6 was removed in c-FLIP$^{-/-}$ MEFs. PP6 knockout efficiency was confirmed by western blotting. Data represent mean ± SD of three independent experiments (Student's t-test ns, not significant).

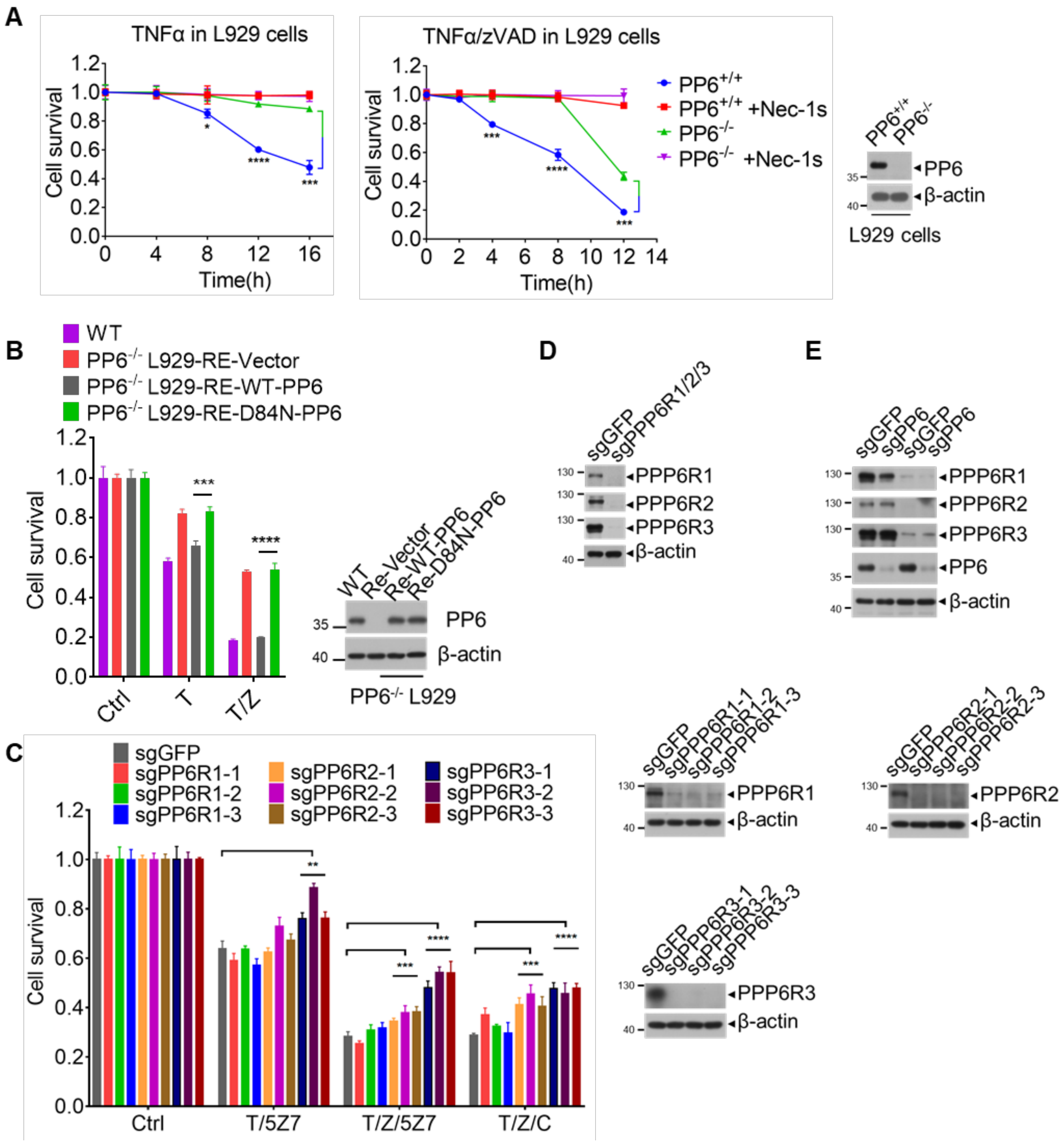

Supplementary Fig. 3 PP6 also promotes TNFα-mediated RIPK1-dependent cell death. A L929 cells were transfected with lentivirus containing sgRNA targeting PP6 to knockout PP6. Cells were pretreated with Nec-1s for 1 h and then treated with TNFα or TNFα/zVAD for indicated periods of time. PP6 knockout efficiency was confirmed by western blotting and

showed on the right panel. **B** PP6$^{-/-}$ L929 cells were reconstituted with control vector, WT-PP6 or phosphatase inactivating mutant D84N-PP6. Cells were treated with TNFα or TNFα/zVAD for 4 h. PP6 reconstitution efficiency was confirmed by western blotting and showed on the right panel. **C** MEFs were transfected with lentivirus containing sgRNAs targeting PPP6R1, PPP6R2 or PPP6R3. Cells were treated with TNFα/5Z-7 and TNFα/zVAD/5Z-7 for 4 h or TNFα/zVAD/CHX for 8 h. The knockout efficiency was confirmed by western blotting and showed on the right panel. **D** PPP6R1/2/3 were triple-knockout in MEFs by specific sgRNAs targeting PPP6R1, PPP6R2 and PPP6R3. The knockout efficiency was confirmed by western blotting. **E** PP6 was further removed in WT or PPP6R1/2/3 triple-knockout MEFs. PP6 knockout efficiency was confirmed by western blotting. Concentrations of reagents used: TNFα, 50 ng/ml; CHX (C), 2 μg/ml; Nec-1s, 10 μM; zVAD (Z), 50 μM; 5Z-7, 300 nM. The cell death in **A**-**C** was measured by CellTiter-Glo assay. Data represent mean ± SD of three independent experiments (Student's t-test *P<0.05, **P<0.01, ***P<0.001, ****P<0.0001).

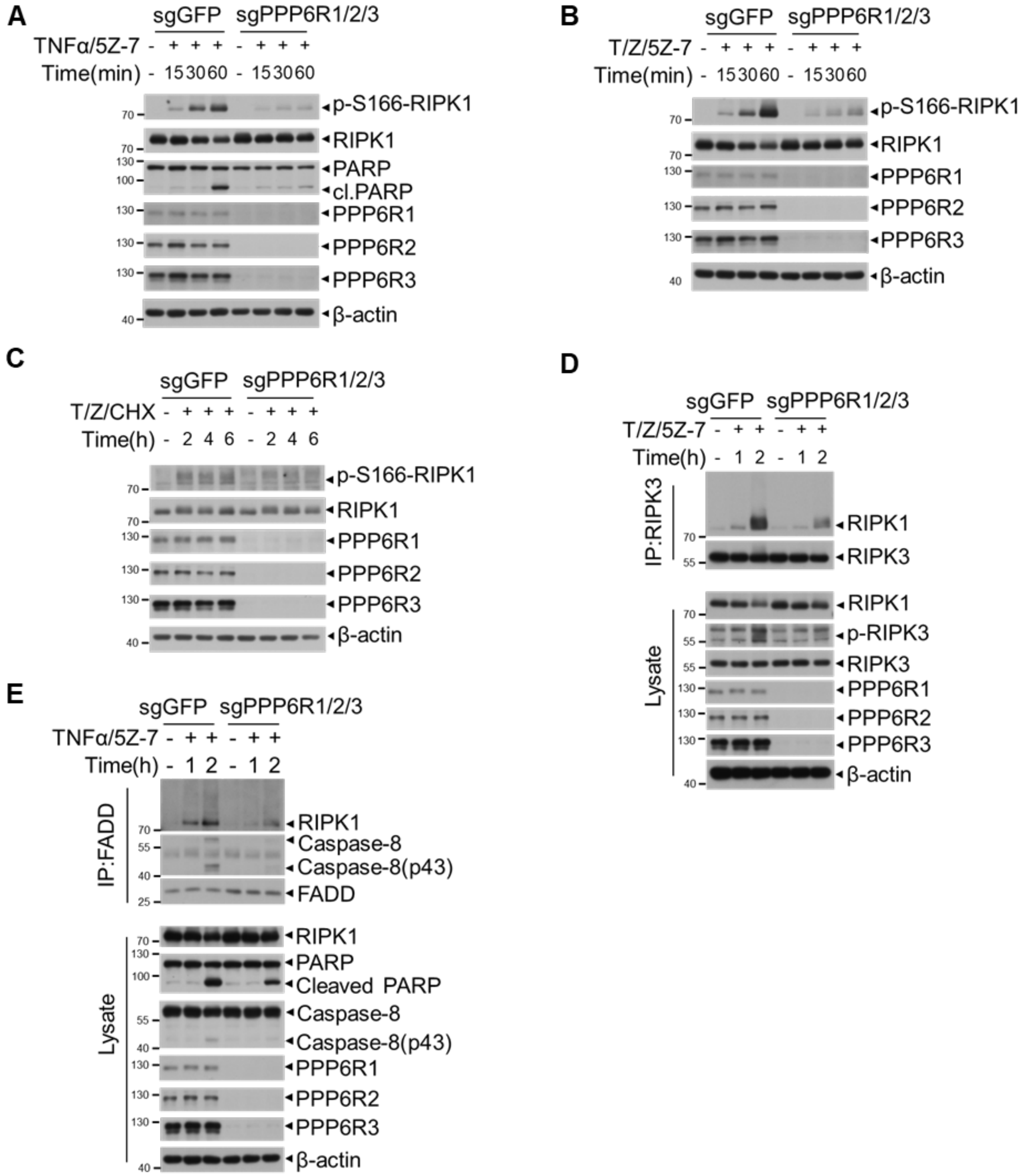

Supplementary Fig. 4 PP6 promotes the activation of RIPK1. A-C WT or PPP6R1/2/3 triple-knockout MEFs were treated with TNFα/5Z-7 and TNFα/zVAD/5Z-7 or TNFα/zVAD/CHX for indicated periods of time. Cells were lysed with RIPA buffer and analyzed by western blotting with indicated antibodies. **D**, **E** WT or PPP6R1/2/3 triple-knockout MEFs were treated with TNFα/zVAD/5Z-7 or TNFα/5Z-7 for indicated periods of

time. Cells were lysed with NP-40 buffer and immunoprecipitated with anti-RIPK3 or anti-FADD antibody. The immunocomplexes and whole-cell lysates were analyzed by western blotting with indicated antibodies. Concentrations of reagents used: TNFα, 50 ng/ml; CHX (C), 2 μg/ml; zVAD (Z), 50 μM; 5Z-7, 300 nM.

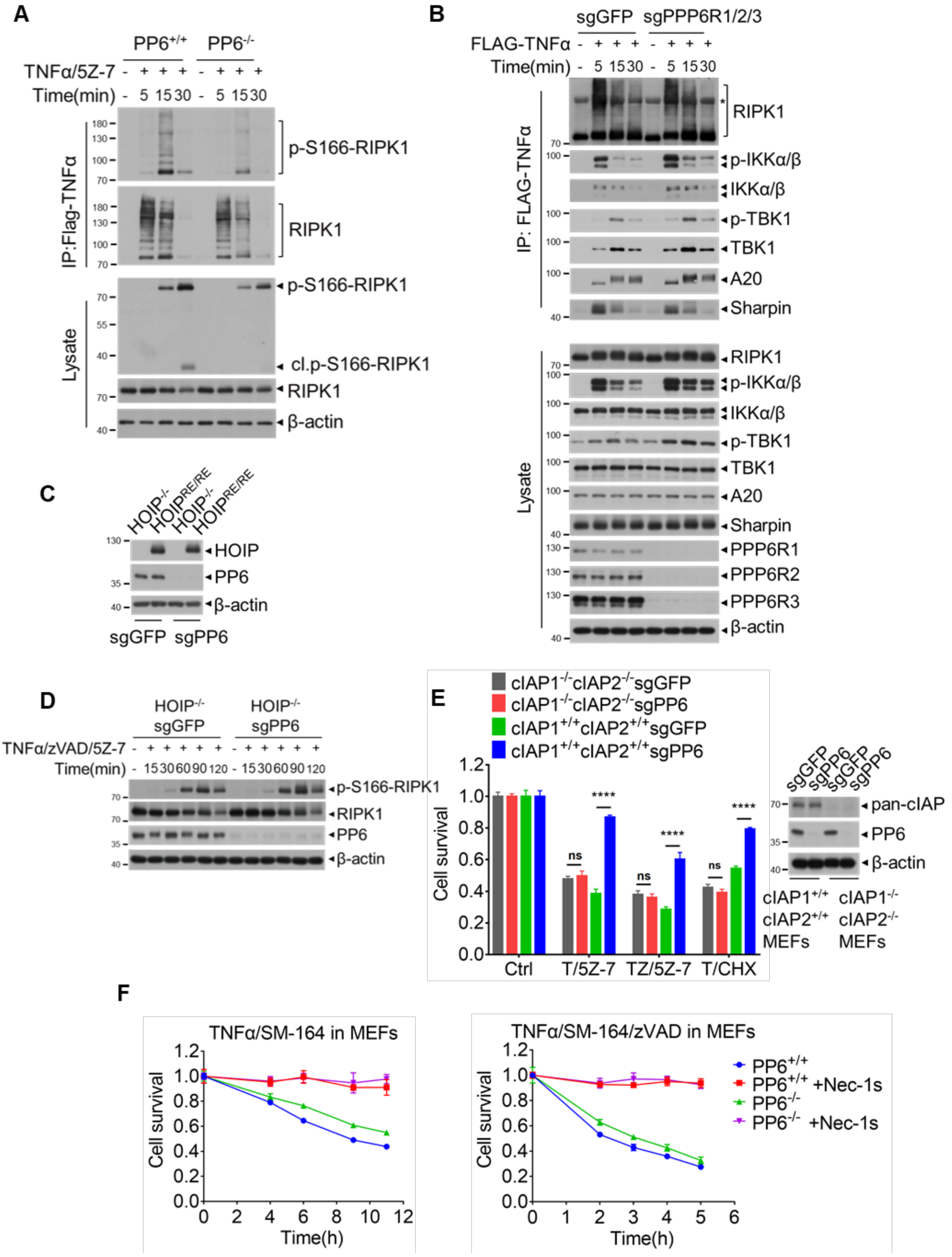
A
PP6+/+
PP6-/-
TNFα/5Z-7
Time(min)
IP:Flag-TNFα
p-S166-RIPK1
RIPK1
Lysate
p-S166-RIPK1
cl.p-S166-RIPK1
RIPK1
β-actin
B
sgGFP
sgPPP6R1/2/3
FLAG-TNFα
Time(min)
IP: FLAG-TNFα
RIPK1
p-IKKα/β
IKKα/β
p-TBK1
TBK1
A20
Sharpin
Lysate
RIPK1
p-IKKα/β
IKKα/β
p-TBK1
TBK1
A20
Sharpin
PPP6R1
PPP6R2
PPP6R3
β-actin
C
HOIP-/-
HOIPRE/RE
HOIP-/-
HOIPRE/RE
HOIP
PP6
β-actin
sgGFP
sgPP6
D
HOIP-/- sgGFP
HOIP-/- sgPP6
TNFα/zVAD/5Z-7
Time(min)
p-S166-RIPK1
RIPK1
PP6
β-actin
E
cIAP1-/-cIAP2-/-sgGFP
cIAP1-/-cIAP2-/-sgPP6
cIAP1+/+cIAP2+/+sgGFP
cIAP1+/+cIAP2+/+sgPP6
Cell survival
Ctrl
T/5Z-7
TZ/5Z-7
T/CHX
ns
****
sgGFP
sgPP6
sgGFP
sgPP6
pan-cIAP
PP6
β-actin
cIAP1+/+ cIAP2+/+ MEFs
cIAP1-/- cIAP2-/- MEFs
F
TNFα/SM-164 in MEFs
Cell survival
Time(h)
TNFα/SM-164/zVAD in MEFs
Cell survival
Time(h)
PP6+/+
PP6+/+ +Nec-1s
PP6-/-
PP6-/- +Nec-1s

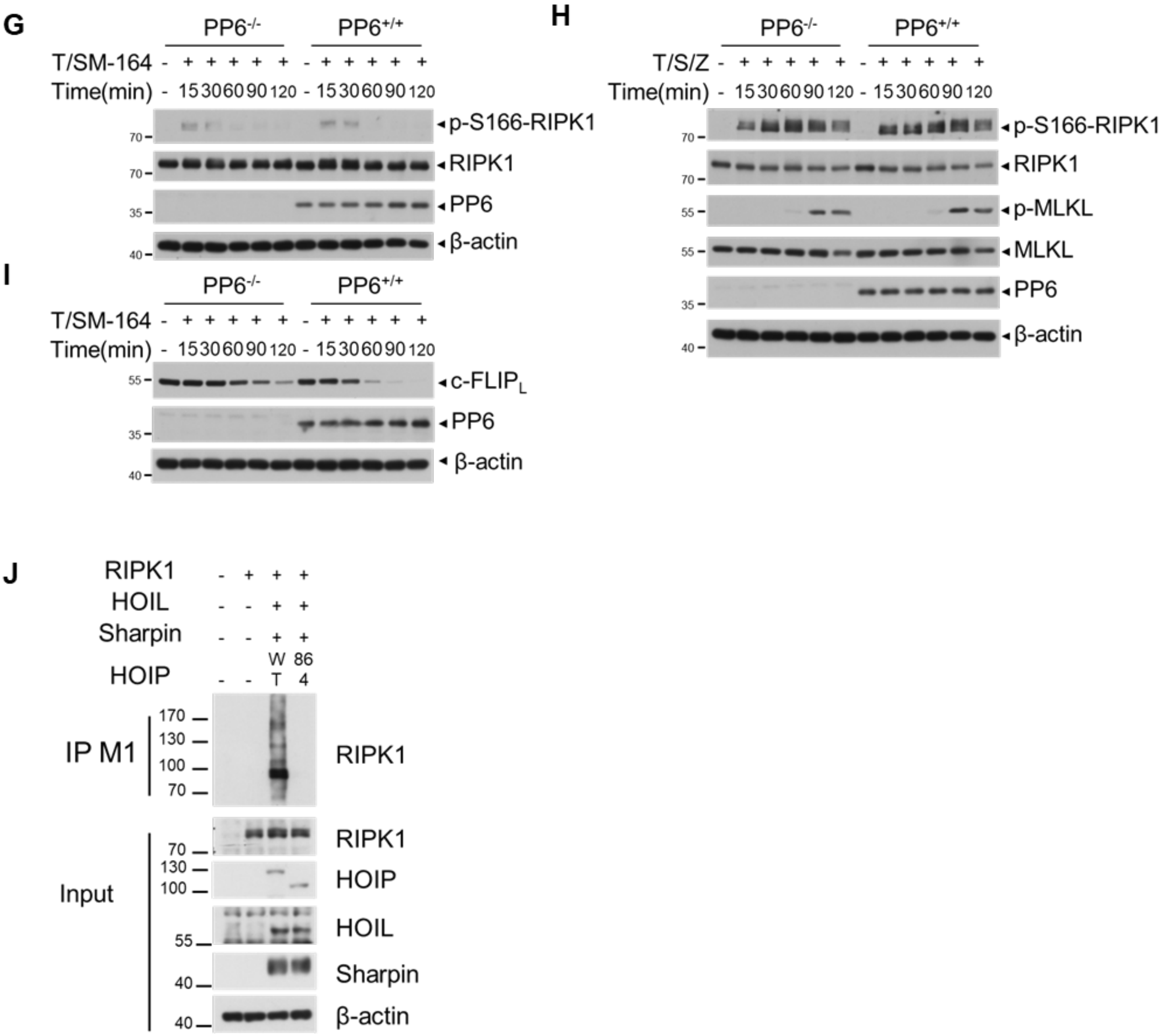

Supplementary Fig. 5 HOIP is required for PP6 to regulate RIPK1 activation and c-FLIP$_L$ degradation. A PP6$^{+/+}$ or PP6$^{-/-}$ MEFs were treated with TNFα/5Z-7 for indicated periods of time. Cells were lysed with NP-40 buffer and immunoprecipitated with anti-Flag beads. The immunocomplexes and whole-cell lysates were analyzed by western blotting with indicated antibodies. **B** WT or PPP6R1/2/3 triple-knockout MEFs were treated with Flag-TNFα for indicated periods of time. Cells were lysed with NP-40 buffer and immunoprecipitated with anti-Flag beads. The immunocomplexes and whole-cell lysates were analyzed by western blotting with indicated antibodies. **C** PP6 was removed in HOIP$^{-/-}$ or HOIP reconstituted MEFs.

PP6 knockout efficiency was confirmed by western blotting. **D** HOIP-/- sgGFP MEFs or HOIP-/- sgPP6 MEFs were treated with TNFα/zVAD/5Z-7 for indicated periods of time. Cells were lysed with RIPA buffer and analyzed by western blotting with indicated antibodies. **E** PP6 was knocked out using CRISPR-Cas9 in WT or cIAP1$^{-/-}$cIAP2$^{-/-}$ MEFs. Cells were treated with TNFα/5Z-7 and TNFα/zVAD/5Z-7 for 2 h or TNFα/CHX for 4 h. PP6 knockout efficiency was confirmed by western blotting and showed on the right panel. **F** PP6$^{+/+}$ or PP6$^{-/-}$ MEFs were pretreated with Nec-1s and SM-164 for 1 h and then treated with TNFα or TNFα/zVAD for indicated periods of time. **G,H** PP6$^{+/+}$ or PP6$^{-/-}$ MEFs were pretreated with SM-164 for 1 h and then treated with TNFα or TNFα/zVAD for indicated periods of time. Cells were lysed with RIPA buffer and analyzed by western blotting with indicated antibodies. **I** PP6$^{+/+}$ or PP6$^{-/-}$ MEFs were pretreated with SM-164 for 1 h and then treated with TNFα for indicated periods of time. Cells were lysed with RIPA buffer and analyzed by western blotting with indicated antibodies. **J** Flag-RIPK1 was co-expressed with LUBAC (HA-HOIP(WT or 1-864 truncation)/Myc-HOIL/Flag-Sharpin) in 293T cells for 20 h. Cells were lysed with urea lysis buffer and immunoprecipitated with chain specific M1 ubiquitin antibody. The immunocomplexes and whole-cell lysates were analyzed by western blotting with indicated antibodies. Concentrations of reagents used: Flag-TNFα, 150 ng/ml; TNFα (T), 50 ng/ml; CHX (C), 2 μg/ml; Nec-1s, 10 μM; zVAD (Z), 50 μM; 5Z-7, 300 nM; SM-164 (S), 1 μM. The cell death in **E** and **F** was measured by CellTiter-Glo assay. Data represent mean ± SD of three independent experiments (Student's t-test ****P<0.0001, ns, not significant).

A

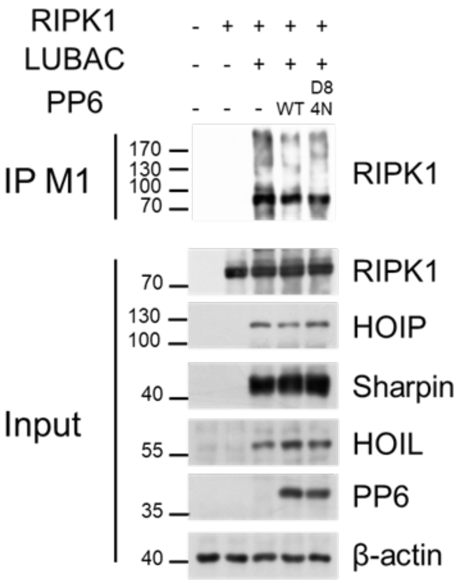

Supplementary Fig. 6 PP6 negatively modulates LUBAC-mediated M1-ubiquitination of RIPK1 and c-FLIP$_L$. A Flag-RIPK1 was co-expressed with LUBAC (HA-HOIP/Myc-HOIL/Flag-Sharpin) and HA-PP6(WT or D84N) in 293T cells for 20 h. Cells were lysed with urea lysis buffer and immunoprecipitated with chain specific M1 ubiquitin antibody. The immunocomplexes and whole-cell lysates were analyzed by western blotting with indicated antibodies.
